# Corrosion casting of the subglottis following endotracheal tube intubation injury: a pilot study in Yorkshire piglets

**DOI:** 10.1186/1916-0216-42-52

**Published:** 2013-10-14

**Authors:** Lukas H Kus, Michael C Sklar, Jaina Negandhi, Marvin Estrada, Antoine Eskander, Robert V Harrison, Paolo Campisi, Vito Forte, Evan J Propst

**Affiliations:** 1 Department of Otolaryngology – Head and Neck Surgery, The Hospital for Sick Children, University of Toronto, 6th Floor, Burton Wing, 555 University Avenue, Toronto, Ontario M5G 1X8, Canada; 2 Laboratory Animal Services, The Hospital for Sick Children, University of Toronto, Toronto, Canada

**Keywords:** Subglottic stenosis, Endotracheal tube injury, Animal model, Corrosion casting, Scanning electron microscopy

## Abstract

**Purpose:**

Subglottic stenosis can result from endotracheal tube injury. The mechanism by which this occurs, however, is not well understood. The purpose of this study was to examine the role of angiogenesis, hypoxia and ischemia in subglottic mucosal injury following endotracheal intubation.

**Methods:**

Six Yorkshire piglets were randomized to either a control group (N=3, ventilated through laryngeal mask airway for corrosion casting) or accelerated subglottic injury group through intubation and induced hypoxia as per a previously described model (N=3). The vasculature of all animals was injected with liquid methyl methacrylate. After polymerization, the surrounding tissue was corroded with potassium hydroxide. The subglottic region was evaluated using scanning electron microscopy looking for angiogenic and hypoxic or degenerative features and groups were compared using Mann–Whitney tests and Friedman’s 2-way ANOVA.

**Results:**

Animals in the accelerated subglottic injury group had less overall angiogenic features (P=.002) and more overall hypoxic/degenerative features (P=.000) compared with controls. Amongst angiogenic features, there was decreased budding (P=.000) and a trend toward decreased sprouting (P=.037) in the accelerated subglottic injury group with an increase in intussusception (P=.004), possibly representing early attempts at rapid revascularization. Amongst hypoxic/degenerative features, extravasation was the only feature that was significantly higher in the accelerated subglottic injury group (P=.000).

**Conclusions:**

Subglottic injury due to intubation and hypoxia may lead to decreased angiogenesis and increased blood vessel damage resulting in extravasation of fluid and a decreased propensity toward wound healing in this animal model.

## Introduction

Subglottic stenosis is a potential complication of endotracheal tube (ETT) intubation. The degree of injury can range from mild mucosal erythema or ulceration to granuloma formation and airway stenosis [1,2]. Injury is believed to be caused by pressure from the ETT on the mucosa overlying the cricoid cartilage [3]. Although the risk of injury usually increases with prolonged intubation, airway injury can occur even within a few hours of intubation and is exacerbated by hypoxic conditions [4,5]. One hypothesis is that when ETT cuff pressure exceeds tissue capillary perfusion pressure, mucosal blood flow is impaired and the resulting edema and ischemic necrosis can lead to formation of fibrotic scar tissue and subglottic stenosis [3,6]. Our group previously developed an animal model of ETT cuff injury that used hypoxia to accelerate the formation of an ischemic subglottic mucosal injury caused by an ETT cuff [7]. We then investigated histopathological changes in the subglottis with varying degrees of ETT cuff pressure and found that constant pressure leads to significant epithelial loss, extensive subepithelial and glandular necrosis, and acute inflammation [7]. Though these studies and others have investigated indirect measures of vascular injury, none have directly examined the microscopic vascular features of the subglottic mucosa following ETT-related injury [3,6].

Corrosion casting is an experimental technique that allows for the study of vascular structures. This method involves the injection of methylmethacrylate, a liquid plastic polymer, into an organ’s blood supply. As the polymer hardens, a three-dimensional cast of the blood vessels is formed. The overlying tissues are then corroded in basic solution and the resulting vascular cast can be analyzed in fine detail using imaging techniques such as scanning electron microscopy (SEM). The purpose of this study was to analyze the microscopic vascular changes that occur in the subglottis following injury secondary to ETT intubation using corrosion casting and SEM.

## Materials and methods

### Animals

This study was approved by the Animal Care Committee at the Hospital for Sick Children. Six Yorkshire piglets (*Sus scrofa domesticus*) were randomized to two groups:

Three control animals were ventilated through a laryngeal mask airway SpO2 = 100%) while undergoing immediate corrosion casting (described below) and three experimental animals were exposed to a 4 hour accelerated intubation injury described elsewhere [7] prior to corrosion casting. The mean age of control piglets was 7.0 +/- 1.0 weeks (range 6–8 weeks) and their mean weight was 14.7 +/- 0.30 kg (range 14.2 - 14.8 kg). The mean age of intubated piglets was 7.0 +/- 0.5 weeks (range 6.5 - 7.5 weeks) and their mean weight was 15.3 +/- 1.67 kg (range 13.4 - 16.4 kg). There was no difference across groups with respect to age or weight (P>.05).

### Accelerated intubation injury model

Animals were intubated and exposed to accelerated ETT cuff injury conditions as described previously elsewhere [3,7,8]. Briefly, animals were sedated with an intramuscular injection of Akmezine (0.2 mL/kg) and were placed under anaesthesia using Isoflurane (5%) delivered by facemask. Animals were intubated with a 6.0 mm or 6.5 mm internal diameter cuffed Sheridan endotracheal tube (ETT; Teleflex Medical, Research Triangle Park, North Carolina) with the cuff directly below the vocal cords and cuff pressure maintained at 25 cm H2O using a Magnehelic manometer (Dwyer Instruments, Michigan City, Indiana). When inflated to 25 cm H2O, the length of contact between the ETT cuff and the subglottic mucosa extended for 2.0 cm or 2.5 cm below the vocal cords for 6.0 mm or 6.5 mm internal diameter Sheridan ETT’s, respectively. All intubations were atraumatic. Animals were placed in the supine position and the ETT was secured to the snout. Ventilation was maintained using a volume-cycled ventilator (Air Shields Ventimeter; Narco Health Company, Hatboro, Pennsylvania). Intravenous fluid was administered through an auricular vein. Hypoxia was induced for 4 hours by ventilating the animal with a mixture of oxygen and nitrous oxide to maintain a target SpO2 of 70% and end-tidal CO2 less than 40 mmHg. Heart rate, respiratory rate, oxygen saturation, end-tidal carbon dioxide levels, and body temperature (rectal) were monitored throughout the procedure. All animals were intubated and maintained under hypoxic conditions by the same investigator (LHK). After 4 hours, the ETT cuff pressure was decreased to 20 cm H2O and the SpO2 was gradually increased to 100% over 15 minutes prior to vascular casting.

### Vascular casting

**Figure 1:**
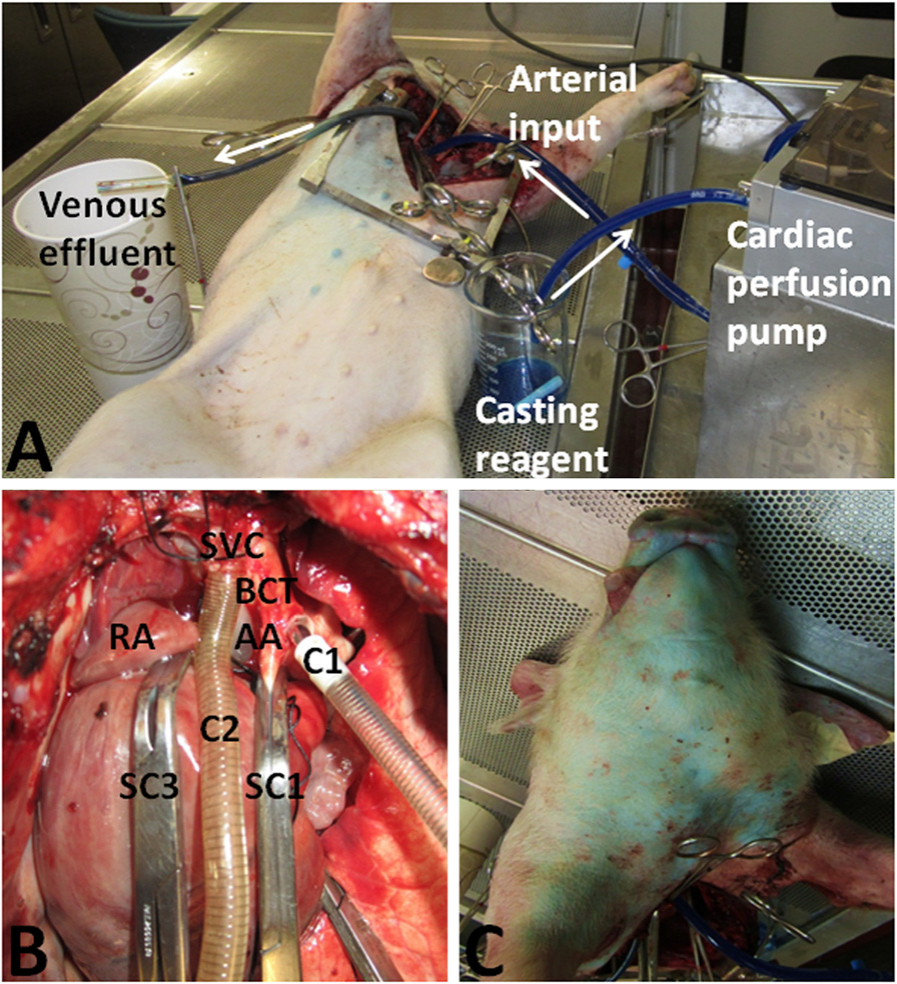
**Creating a vascular circuit in the head and neck for corrosion casting. (A)** Blue casting reagent is retrieved from a reservoir by a cardiac perfusion pump and introduced into the arterial circulation via an aortic cannula. After circulating through the head and neck, the venous effluent is collected via a cannula in the superior vena cava. White arrows indicate direction of fluid flow. **(B)** A Satinsky clamp (SC1) is placed on the ascending aorta (AA) proximal to the bicarotid trunk (BCT). Another is placed distal to the bicarotid trunk on the descending aorta (not seen). A third Satinsky clamp (SC3) is placed on the superior vena cava (SVC) just proximal to the right atrium (RA). One cannula (C1) is placed in the bicarotid trunk (BCT) and another (C2) is placed in the superior vena cava (SVC). **(C)** A change in skin colour from pink to blue denotes complete perfusion of casting reagent.

A 25 cm vertical thoracotomy incision was made in the midline of the chest from sternal notch to xiphoid process using monopolar cautery and Liston bone cutting forceps. The superior vena cava and aortic arch were dissected free from their posterior attachments and the pig was euthanized with an intracardiac injection of Euthanyl (25 mg/kg). The mean duration of surgery was 97.3 +/- 8.9 minutes (range 85–113 minutes). Contrary to humans, whereby the left common carotid artery arises from the aorta and the right common carotid artery arises from the brachiocephalic artery, pigs have a solitary bicarotid trunk that divides distally into both left and right common carotid arteries [9]. Satinsky clamps were placed on the aortic arch proximally and distally to the bi-carotid trunk as well as on the superior vena cava adjacent to the right atrium. This created a vascular circuit from the bi-carotid trunk to the superior vena cava (Figure 1). Purse string sutures were placed in the superior vena cava and the arch of the aorta at the takeoff of the bi-carotid trunk using 5–0 Polypropylene sutures (Ethicon, Somerville, New Jersey). These sites were incised with a scalpel and cannulated with 20 French aortic and venous catheters (Terumo Cardiovascular Systems, Ann Arbor, Michigan), respectively (Figure 1). Choker sleeves were used to tighten the purse string sutures and minimize leakage from the cannulation sites. The aortic cannula was attached to a pediatric cardiac perfusion pump (Medtronic, Minneapolis, Minnesota) using ¼ inch surgical tubing (Saint-Gobain Performance Plastics, Akron, Ohio). One-quarter inch surgical tubing was also attached to the venous cannula and was left open to air to allow the intravascular effluent to drain out. This created a vascular loop through the animal’s head and neck whereby fluid could be injected and recovered (Figure 1). The vascular loop was flushed with 3 L of heparinized (15 units/mL) 0.9% saline at a rate of 150 mL/min until there was no more blood in the circuit (i.e. venous cannula effluent was clear). Arterial and venous cannulae were clamped and the animals were transported to a ventilated room for casting.

Casting reagent consisted of 200 mL Batson’s #17 monomer base solution (Polysciences, Warrington, Pennsylvania), 80 mL ProBase Cold Monomer (Ivoclar Vivadent, Schaan, Liechtenstein), 30 mL Batson’s #17 catalyst (Polysciences, Warrington, Pennsylvania), 24 drops Batson’s #17 promoter (Polysciences, Warrington, Pennsylvania), and 3 g phthalocyanine blue dye (Polysciences, Warrington, Pennsylvania). This blue casting reagent (310 mL) was perfused into the aortic cannula until it was seen extravasating from the venous cannula, indicating that it had perfused the entire head and neck. The animal’s skin colour changed from pink to blue indicating complete perfusion to the capillary level (Figure 1). The animal was placed on ice for 3 hours while the casting resin solidified.

### Corrosion

**Figure 2:**
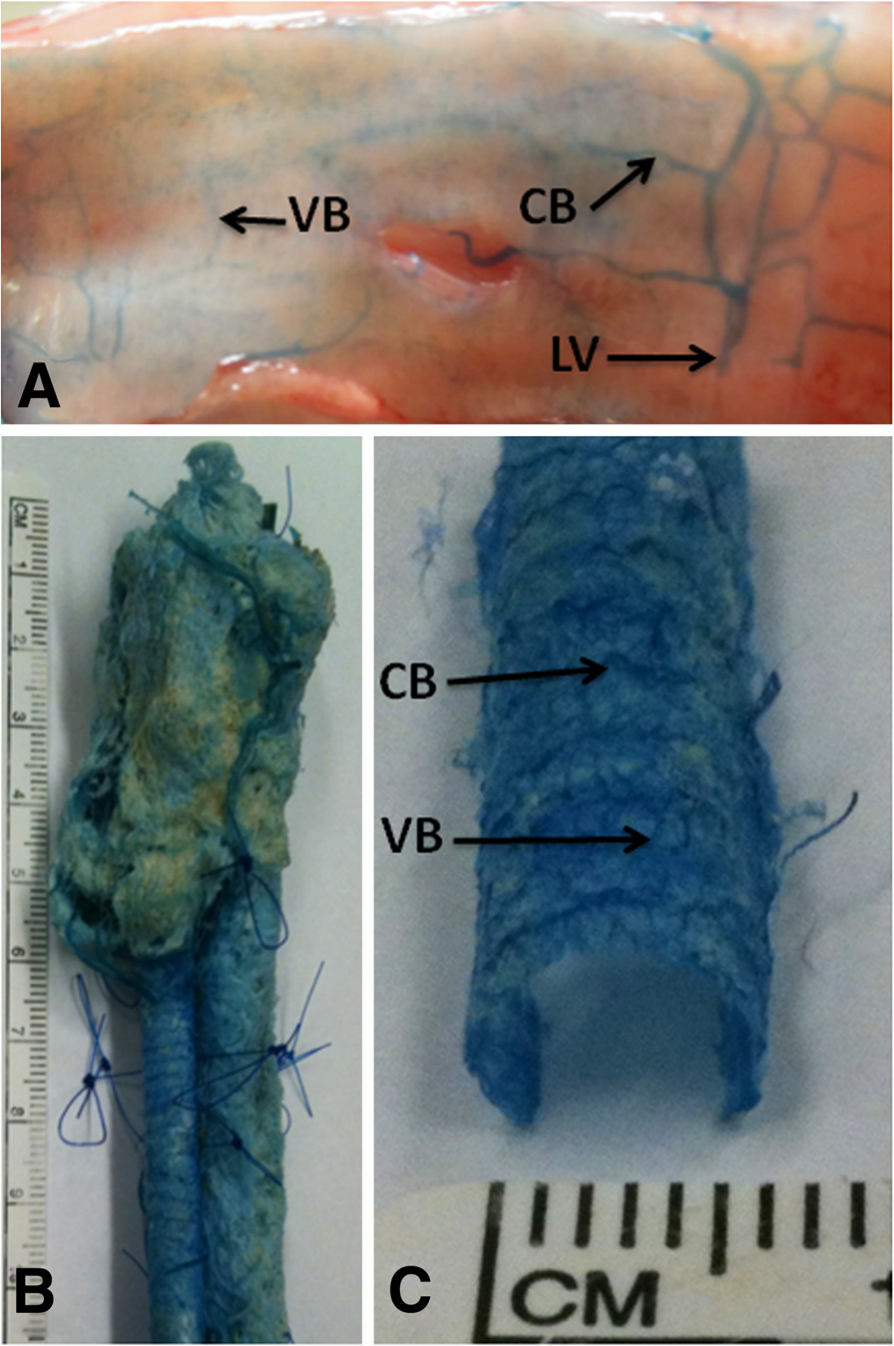
**Gross features of normal tracheal vascular anatomy. (A)** Anterior and lateral lumenal surface of casted but uncorroded trachea (opened posteriorly and flattened). Longitudinal vessels (LV) give off circumferential branches (CB) that are connected by vertical branches (VB). **(B)** Corroded specimen showing thyroid cartilage, trachea and esophagus with sutures delineating important anatomical areas. **(C)** Closer view of casted specimen showing circumferential branches (CB) and vertical branches (VB).

An *en bloc* section of upper airway tissue from the superior aspect of the thyroid cartilage to the carina was harvested. The inferior border of the true vocal cords, the inferior border of the cartilaginous graft and every three tracheal rings inferiorly were marked with 5-O Nylon sutures (Ethicon, Somerville, New Jersey) because they are resistant to corrosion. The specimen was sutured to a 6 mm diameter white polypropylene drinking straw for structural support and ease of vessel visualization. The specimen was then corroded in a 16% KOH bath at 45°C for 2 days, washed three times in distilled water, decalcified in a 2% HCl bath at 45°C for 1 day, then washed a further three times in distilled water. This cycle was repeated as needed until the specimen was cleared of residual tissue, fat, and cartilaginous debris (Figure 2).

### Scanning Electron Microscopy (SEM)

**Figure 3:**
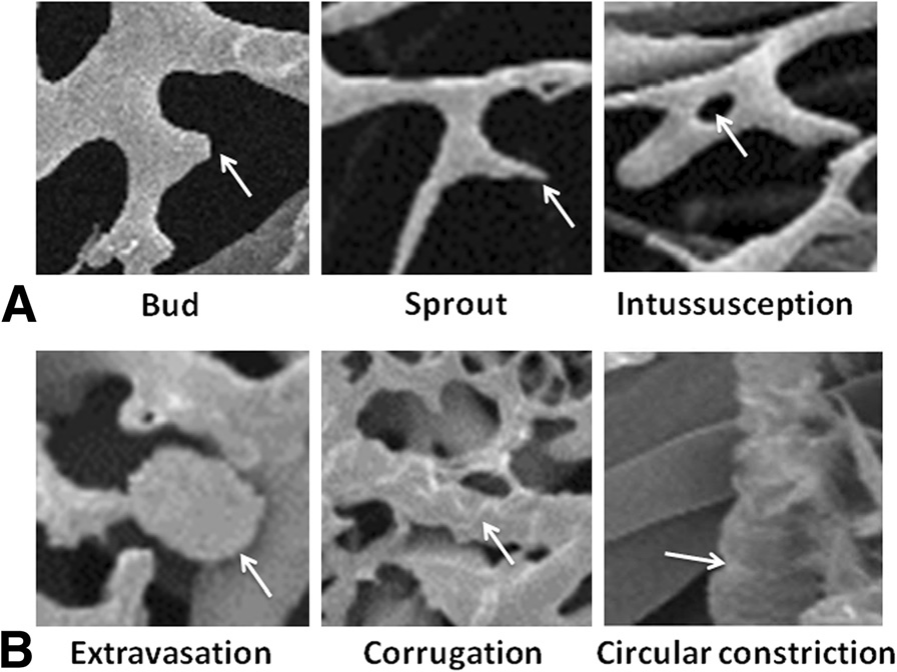
**SEM imaging of vascular features. (A)** Angiogenic and **(B)** hypoxic/degenerative features.

Airway casts were freeze-dried overnight in a Micro Modulyo freeze dryer (Thermo Electron, Marietta, Ohio) and divided vertically along their posterior aspect to visualize the anterior lumen of the trachea. An area of anterior tracheal cast 2.0 or 2.5 cm distal to the true vocal cords (depending on whether 6.0 or 6.5 ETT used, respectively) was excised and mounted on a SEM stub with the lumen side facing upward. Specimens were sputter coated with gold and viewed using SEM (Hitachi S570, Tokyo, Japan) under constant conditions (accelerating voltage 5 kV, magnification 190X, working distance 18,400 μm). Six images were obtained by one author (JN) for each animal from a randomly selected area within the defined sample region. Image identifiers were removed, and images were coded, randomized and presented to two blinded observers (LHK, MS), each of whom counted features in every image (or field of view). Angiogenic features [10] (budding, sprouting, and intussusception) and hypoxic or degenerative features [11] (resin extravasation, corrugations, and circular constriction) were recorded for each field of view (FOV; Figure 3).

### Statistics

Data were analyzed using SPSS version 21 (IBM, Armonk, New York). Based on analysis for normality, equality of variance and the relatively small sample size, non-parametric descriptive statistics were used (median and inter-quartile range).

Comparisons between control and experimental groups was performed using Mann–Whitney U (aka Wilcoxon Rank Sum) test. Within a given group, comparison of two imaging features was performed using the related-samples Wilcoxon Signed Rank test and comparison of three imaging features was performed using the related-samples Friedman's 2-way analysis of variance (ANOVA) by ranks. The limit of significance was taken to be 0.625% (p<0.00625) for all comparisons after Bonferroni correction for multiple comparisons. Interrater reliability was assessed using intra-class correlation (ICC) coefficient with an ICC greater than 0.8 considered acceptable (high reliability).

## Results

### Gross and microscopic vascular anatomy of control subglottis and trachea

**Figure 4:**
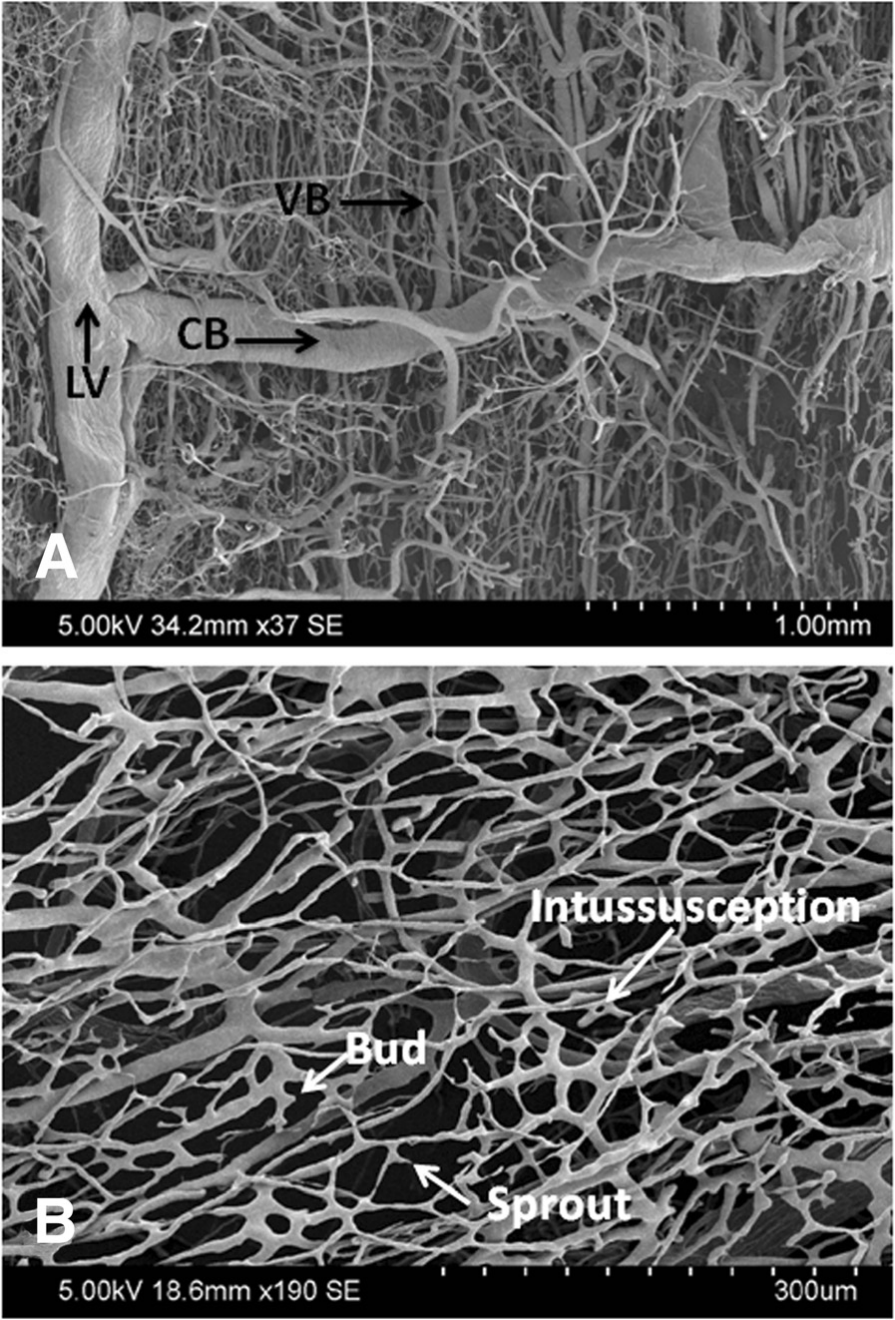
**SEM imaging of corrosion casted control trachea. (A)** External surface showing longitudinal vessels (LV) that give off circumferential branches (CB) connected by vertical branches (VB) that course deeply to form a dense capillary plexus. **(B)** The lumenal surface is an interconnected mesh of capillaries with vessel buds, sprouts, and intussusceptions. Hypoxic or degenerative features are not visible.

**Figure 5:**
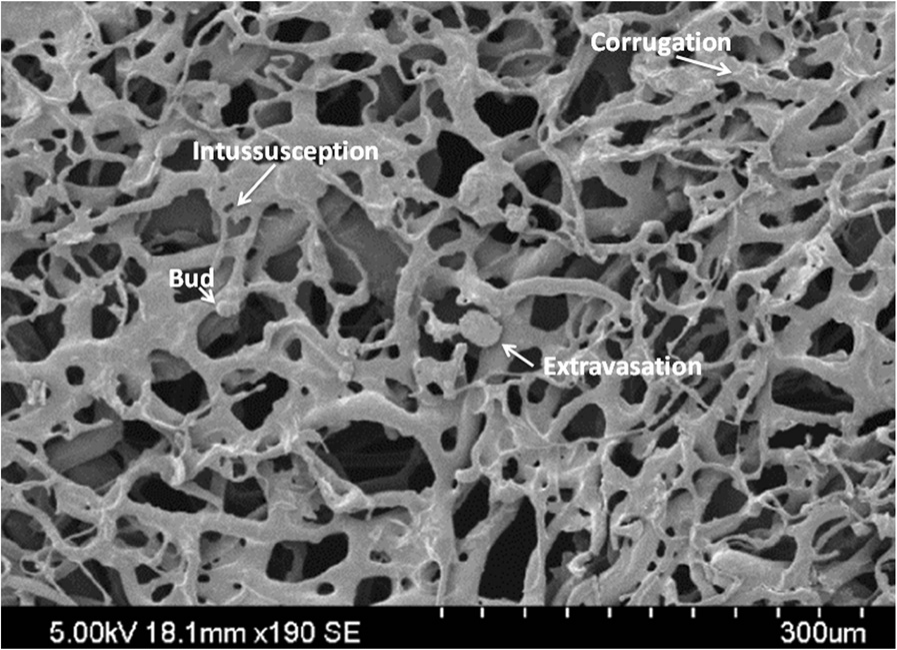
**Gross and microscopic appearance of subglottic corrosion casts after hypoxic endotracheal tube cuff injury. (A)** SEM image of subglottic lumenal surface showing an interconnected mesh of capillaries. Vessel buds, intussusceptions, resin extravasations, and vessel corrugations are seen (other angiogenic and hypoxic or degenerative features are not visible).

All 3 experimental animals survived hypoxic conditions and corrosion casting was successful in all 6 animals. Gross inspection of casts from control animals revealed the subglottic and tracheal vasculature to be cylindrical, measuring approximately 0.3 mm in thickness (Figure 2). There were two large longitudinal vessels (LV, one on each side) at the posterior aspect of the trachea near the tracheoesophageal groove (Figure 2). Regularly-spaced circumferential branches (CB) coursed between the longitudinal vessels which interdigitated with the dense vasculature of the esophagus posteriorly and anastomosed anteriorly in the midline of the trachea (Figure 2). Smaller vertical branches (VB) connected the circumferential vessels and travelled deeply to form a dense plexus of fine capillaries on the lumenal side of the specimen. SEM of the posterior surface of the trachea demonstrated this complex vascular network of longitudinal, circumferential and vertical vessels in better detail (Figure 4), which was vastly different in appearance than the vascular network on the lumenal surface that demonstrated a fairly uniform interconnected mesh of capillaries (Figure 5).

### Quantitative analysis of control and accelerated subglottic injury animals

**Figure 6:**
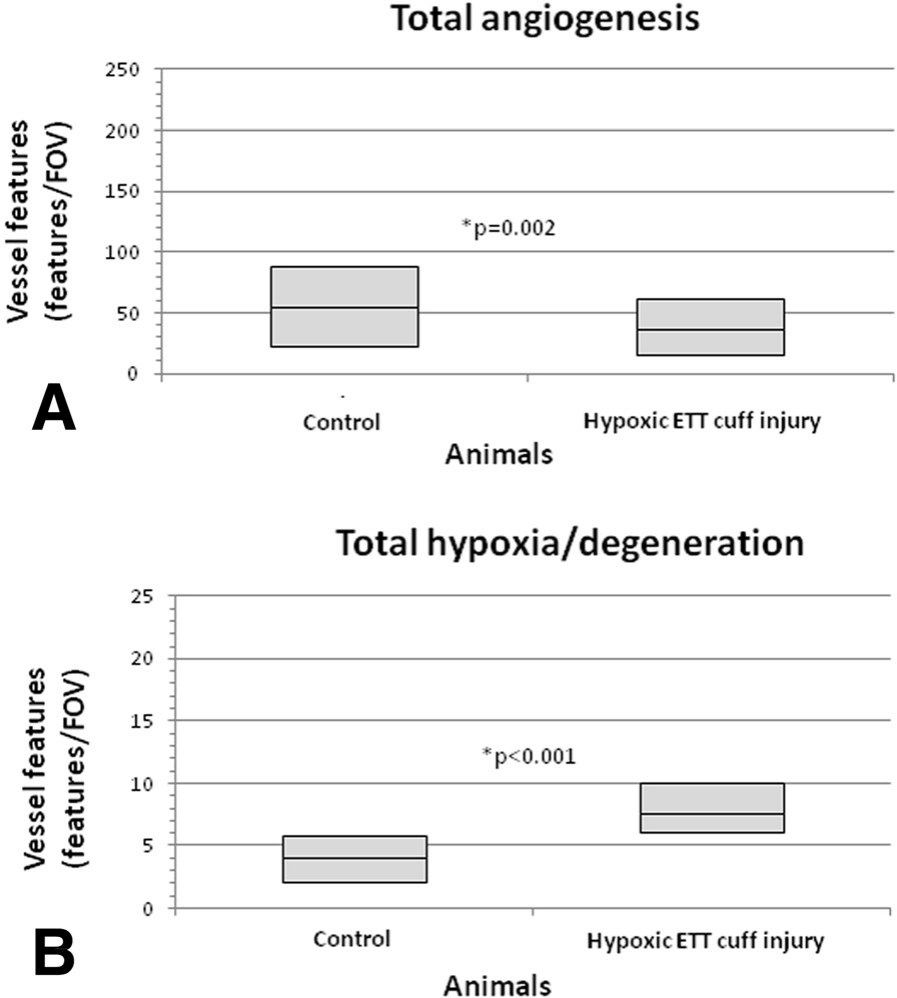
**Box plots comparing total aggregate vascular features. (A)** Total angiogenesis includes vessel budding, sprouting, and intussusceptions. **(B)** Total hypoxic/degenerative features includes extravasations, corrugations, and circular constrictions. Median values are displayed as a line within a box, the borders of which denote the inter-quartile range. Asterisks indicate significance levels.

Results are outlined in Table 1. Animals in the accelerated subglottic injury group had less overall angiogenic features (P=.002) and more overall hypoxic/degenerative features (P=.000) compared with controls (Figure 6). Amongst angiogenic features, there was decreased budding (P=.000) and a trend toward decreased sprouting (P=.037) in the accelerated subglottic injury group with an increase in intussusception (P=.004). Amongst hypoxic/degenerative features, extravasation was the only feature that was significantly higher in the accelerated subglottic injury group (P=.000). The total degree of sprouting-type angiogenesis (budding and sprouting) was higher than intussusceptive angiogenesis in both controls and accelerated subglottic injury animals. The overall intra-class correlation coefficient for the two observers counting vessels was .999, indicating very high reliability.

**Table 1:** Angiogenic and hypoxic/degenerative features in the subglottis of control and intubated animals

	**Control group (Features/FOV)**	**Intubation group (Features/FOV)**	**Significance**
**Vessel features**	**Median (IQR)**	**Median (IQR)**	**P value**
**Angiogenic**			
** Total angiogenic**	66.0 (54.5–81.0)	46.5(36.0–56.0)	.002
** Budding**	33.5 (28.3–43.0)	16.0 (13.3–23.3)	.000
** Sprouting**	30.0 (22.7–39.3)	20.5 (18.0–25.0)	.037
** Intussusception**	1.5 (0.3–2.7)	4.5 (3.3–6.7)	.004
**Hypoxic/Degenerative**			
** Total hypoxic/degenerative**	4.0(2.0–5.8)	7.5(6.0–10.0)	.000
** Extravasation**	1.5 (1.0–3.7)	5.5 (4.0–7.7)	.000
** Corrugation**	1.0 (0–2.0)	1.0 (0–1.0)	.963
** Circular constriction**	0 (0–1.0)	1.0 (0–2.7)	.079

SD = standard deviation.

IQR = interquartile range.

FOV = field of view (one image on electron microscopy).

## Discussion

Endotracheal intubation is a life-saving measure that can cause varying degrees of injury to the subglottis, ranging from mild mucosal ulceration to complete stenosis [1,2]. Subglottic stenosis is believed to result from ETT cuff pressures that exceed capillary perfusion pressure leading to ischemia, necrosis, perichondritis, chondritis, and fibrotic scar formation [3,12]. Hypoxia is also believed to predispose subglottic mucosa to injury [4,13]. While the link between ETT cuff pressure and mucosal necrosis is well established, the precise vascular etiology of these changes is not clearly understood. The purpose of the present study was to analyze the microscopic vascular changes that occur in the subglottis following injury secondary to ETT intubation using corrosion casting and SEM.

The gross vascular anatomy of control tracheas in our study revealed two large longitudinal vessels (LV) on either side of the posterior aspect of the trachea, regularly-spaced circumferential branches between the longitudinal vessels, and smaller vertical branches connecting the circumferential vessels that penetrated deeply to form a dense plexus of fine capillaries on the lumenal side. Even though our study is the first to describe this vascular network in pigs, it has been described previously in human fetuses, guinea pigs, sheep, and dogs [14-17]. The regularly spaced circumferential branches in humans traverse the intercartilaginous spaces, where they provide small branches to the superficial perichondrial vascular bed and then further ramify to pierce the tracheal wall and supply the microcirculation of its mucosal lining [14]. Anatomical similarities between porcine and human tracheal vasculature suggest that results from our study may be translatable to human patients.

Animals in the accelerated subglottic injury group had less overall angiogenic features (P=.002) compared with controls. Amongst angiogenic features, there was decreased budding (P=.000) and a trend toward decreased sprouting (P=.037) in the accelerated subglottic injury group with an increase in intussusception (P=.004)Angiogenesis occurs in two ways, namely sprouting and intussusception. In sprouting angiogenesis, existing blood vessels develop a bud or outgrowth that narrows and extends into a new branch or sprout [18]. This occurs by proteolytic degradation of extracellular matrix followed by chemotactic migration of endothelial cells, formation of a lumen, and endothelial maturation [19]. In intussusceptive angiogenesis, a pre-existing vascular plexus divides internally into mature capillaries. This phenomenon is mediated by bridges of interstitium that widen until a perforation forms through their center [20]. These perforations elongate until a groove is formed between two adjacent rows of endothelial cells, creating the walls of two distinct capillary vessels [20]. The dominant type of angiogenesis varies by organ, although both types may occur concurrently [19]. Maturation of the vasculature involves pruning of the newly formed vascular tree and remodeling of blood vessels [19]. Interestingly, results from the present study demonstrated both sprouting and intussusceptive angiogenesis in control and experimental piglets, suggesting continuous revascularization even without being subjected to injury. This may be a feature of tracheas in general or may be due to the rapid growth of the *Sus scrofa* piglet trachea at a young age. The finding that animals in the accelerated subglottic injury model showed fewer angiogenic features of budding and sprouting with increased intussusceptions than controls suggests an attempt at rapid revascularization. Angiogenesis is required to restore oxygenation and allow new tissue to grow to fill a wound space. Decreased angiogenesis likely leads to delayed growth of new tissue over the wounded area of subglottis, leaving cartilage exposed and at risk for infection, granulation and scar formation.

Animals in the accelerated subglottic injury group had more overall hypoxic or degenerative features than controls (P<.001). However, extravasation was the only feature that attained significance (P<.001). Extravasations, circular constrictions and corrugations have been found in corrosion casts of brains of rats subjected to cerebral ischemia [11]. Extravasations were thought to reflect damaged microvessels that leaked plasma or incurred a small hemorrhage, and these increased in frequency with increasing duration of ischemia. A greater number of resin extravasations in our accelerated subglottic injury group suggests that the subglottic injury likely resulted from vascular injury leading to extravasation of fluid from leaky blood vessels in the form of edema or hemorrhage. Inflammatory mediators released in this extravasated fluid could contribute to subglottic injury. Corrugations are thought to be related to vasospasm caused by convolutions of the internal elastic lamina in constricted arterioles and are not seen in capillaries due to their decreased contractility compared to arterioles [11,21,22]. Circular constrictions are also believed to be due to vasoconstriction and have been seen in rat studies investigating cerebral hemorrhage and vasoconstrictive neurotransmitters [21,22]. The present study found no significant difference across groups with respect to circular vessel constrictions or corrugations. One possible explanation is that vasospasm does not play a major role in subglottic injury caused by an ETT cuff. Alternatively, the casting procedure itself may have negated these vascular changes. However, circular constrictions and corrugations were seen in both control and experimental groups, supporting the former hypothesis.

The main limitation of our study is its small sample size and the limited number of angiogenic and hypoxic or degenerative features that can be counted visually. In addition, artifacts from corrosion casting could have skewed the results. However, such artifacts would not likely preferentially affect one group over the other. Future studies investigating a larger number of animals and a greater variety of vascular features using computerized software may yield more detailed results. Future studies investigating the role of mechanical or pharmacological interventions in preventing vascular injury in the subglottis are required.

## Conclusion

We studied the role of vascular injury in subglottic stenosis using an animal model of accelerated subglottic intubation injury in combination with corrosion casting and SEM imaging. Results suggest that subglottic injury due to intubation and hypoxia may lead to decreased angiogenesis and increased blood vessel damage resulting in extravasation of fluid and a decreased propensity toward wound healing in this animal model. Future studies investigating a larger number of animals subjected to a prolonged period of intubation are required.

## Competing interests

The authors declare that they have no competing interests.

## Authors’ contributions

LHK carried out the experiment and drafted the manuscript. MCS evaluated photographs for features described and critically appraised the manuscript. JN carried out electron microscopy. ME assisted with the experiment. AE assisted with the statistical analysis and critically appraised the manuscript. RVH assisted with electron microscopy and critically appraised the manuscript. PC critically appraised the manuscript. VF critically appraised the manuscript. EP conceived and supervised the experiment and drafted the manuscript. All authors read and approved the final manuscript.

## Special Note

This manuscript was presented at the Poliquin competition at the 67^th^ Canadian Society of Otolaryngology Annual Meeting, Banff, Alberta, June 2–4, 2013.
